# The stay strong app as a self-management tool for first nations people with chronic kidney disease: a qualitative study

**DOI:** 10.1186/s12882-022-02856-x

**Published:** 2022-07-09

**Authors:** Tricia Nagel, Kylie M. Dingwall, Michelle Sweet, David Kavanagh, Sandawana W Majoni, Cherian Sajiv, Alan Cass

**Affiliations:** 1 Menzies School of Health Research, Charles Darwin University, Casuarina, PO Box 41096, Darwin, NT 0811 Australia; 2 Menzies School of Health Research, Charles Darwin University, Alice Springs, NT 0870 Australia; 3 Centre for Children’s Health Research and School of Psychology & Counselling, Faculty of Health, Queensland University of Technology (QUT), Brisbane, QLD 4101 Australia; 4 Department of Nephrology, Royal Darwin Hospital, Northern Territory Department of Health, Darwin, NT 0810 Australia; 5 Northern Territory Medical Program, Flinders University, Darwin, NT 0815 Australia; 6 Central Australian Renal Services, Alice Springs Hospital, Northern Territory Department of Health, Alice Springs, NT 0870 Australia

**Keywords:** Self-management, Chronic kidney disease, Indigenous, Wellbeing, First nations

## Abstract

**Background:**

The high burden of chronic kidney disease in First Nations peoples requires urgent attention. Empowering people to self-manage their own condition is key, along with promotion of traditional knowledge and empowerment of First Nations communities. This study explores the potential of a culturally responsive tool, already found to have high acceptability and feasibility among First Nations people, to support self-management for First Nations people with kidney failure. The Stay Strong app is a holistic wellbeing intervention. This study explores the suitability of the Stay Strong app to support self-management as shown by the readiness of participants to engage in goal setting. Data were collected during a clinical trial which followed adaption of research tools and procedures through collaboration between content and language experts, and community members with lived experience of kidney failure.

**Methods:**

First Nations (i.e., Aboriginal and Torres Strait Islander) participants receiving haemodialysis in the Northern Territory (*n* = 156) entered a three-arm, waitlist, single-blind randomised controlled trial which provided collaborative goal setting using the Stay Strong app at baseline or at 3 months. Qualitative data gathered during delivery of the intervention were examined using both content and thematic analysis.

**Results:**

Almost all participants (147, 94%) received a Stay Strong session: of these, 135 (92%) attended at least two sessions, and 83 (56%) set more than one wellbeing goal. Using a deductive approach to manifest content, 13 categories of goals were identified. The three most common were to: ‘connect with family or other people’, ‘go bush/be outdoors’ and ‘go home/be on country’. Analysis of latent content identified three themes throughout the goals: ‘social and emotional wellbeing’, ‘physical health’ and ‘cultural connection’.

**Conclusion:**

This study provides evidence of the suitability of the Stay Strong app for use as a chronic condition self-management tool. Participants set goals that addressed physical as well as social and emotional wellbeing needs, prioritising family, country, and cultural identity. The intervention aligns directly with self-management approaches that are holistic and prioritise individual empowerment. Implementation of self-management strategies into routine care remains a key challenge and further research is needed to establish drivers of success.

## Background

Chronic kidney disease is one of the most important causes of increasing burden of disease globally [1]. Conditions such as kidney disease are closely linked to major noncommunicable diseases. The World Health Organization (WHO) Global Action Plan for the Prevention and Control of Noncommunicable Diseases aims to reduce the burden of these diseases by 2025 [2]. The Plan highlights that health disparities exist between First Nations peoples and non-First Nations populations in incidence and common risk factors, and that these disparities are often linked to historical, economic and social factors. These factors include colonial policies and practices where health systems and models of care were shaped for dominant society, and were not contextualised for First Nations communities [3]. Most First Nations populations in colonised countries experience poor health outcomes relative to their non-First Nations counterparts [4, 5]. The health challenges that First Nations peoples face are notably similar the world over, in Australia, New Zealand, the United States, circumpolar northern nations and low- and middle-income countries. These challenges are deeply rooted in social disparities [6–8]. The WHO encourages the involvement of First Nations peoples and communities in policies and programs, and recognition and promotion of cultural heritage and traditional knowledge. Prioritising the involvement, collaboration and empowerment of First Nations communities and leadership is deemed critical to successful transformation of healthcare [2].

In Australia, people in remote and very remote areas such as the Northern Territory (NT) experience much higher rates of kidney disease [9, 10]. The NT has the highest proportion of First Nations residents among its population—an estimated 31% (78,600 people) in 2020 [11]. The NT also has the highest incidence and prevalence of chronic kidney disease requiring kidney replacement therapy including haemodialysis, peritoneal dialysis or kidney transplantation [12]. Furthermore, age adjusted prevalence for the NT First Nations population is up to 17 times that of the non-First Nations population [13, 14]. The link between increasing disease burden and health expenditure highlights the need to explore new, more effective intervention strategies. For example, recent research has shown that surprisingly, the cost of relocating people to urban areas for dialysis may be greater than the cost of remote service delivery [15]. The need for new approaches is also driven by the high societal cost and impact on individual quality of life [16].

Approaches to managing chronic illness are shifting from the traditional provider–patient relationship to shared decision making and a more collaborative partnership with health care providers. Chronic conditions have common management challenges including dealing with symptoms and disability; managing complex medication regimens; maintaining nutrition, diet, and exercise, lifestyle adjustments; and engaging in effective interactions with health care providers. Addressing these challenges underpin the focus away from direct patient care toward self-management [17]. Patient centred care approaches [18], self-management support [19], use of patient navigators [20] and peer led support [21] are among a range of strategies outside of direct patient care that seek to address quality of life of chronically ill patients and the concurrent demand for services.

Based on the Chronic Care Model, self-management is defined as ‘the degree to which a patient with a chronic condition is able and willing to control his or her daily life.’ Models of self-management in practice vary widely from provider-set goals to individual empowerment in goal setting, from a biomedical to a holistic focus, and from individual to group to system-wide interventions [22, 23]. Common elements of current models include increased patient participation in care, collaborative goal-setting, and planning of treatment [24]. Patient-centred care and self-management approaches have been identified as having significant potential to result in positive impact for patients with chronic diseases. Patient-centred care is attentive to patients’ psychosocial as well as physical needs and encourages shared control of the consultation and management of health issues [25].

There is increased awareness of the need to promote conceptual clarity regarding self-management and its integration into clinical practice. The common assumption that the self-manager is always a ‘patient’ has been challenged. ‘Person’ centred care is a preferred concept and term that acknowledges the broader context of individuals with chronic conditions which exists outside of clinical settings [26]. Self-management proponents recognise that many decisions are made by people in their day to day lives, away from both the scrutiny and the support of health care providers. These decisions influence their illness trajectory and are impacted by self-management skills. In this sphere of day to day challenges and solutions, the person is the expert [27]. Hence, self-management of chronic illness does not exist in a vacuum, but rather within the context of other people and influences, including friends, family members and community [28]. This view is supported by findings that individuals with higher levels of family support have greater adherence to self-management and better control over their conditions [17].

McWilliam et al. (2009) contrast patient centred care with the ‘empowerment partnering’ approach. The empowering partnering approach replaces a traditional medical care paradigm with a broader ‘health’ paradigm. It engages the person with chronic illness in pursuing life-related goals and managing the illness experience. It thus potentially fulfils fundamental social needs and ensures that the individual’s subjective will and feelings are incorporated into care management strategies [29]. Health care structures must also shift toward support of individuals to manage their conditions in their own way ‘outside’ the health system, promoting empowerment and health equity [26]. This view is reflected in social ecology models which see chronic illness as arising from the interplay of influences within a complex system (from the genome to the macro-environment) and their change over time. Social ecology approaches generally favour diversity and adaptation of programs to meet the individual and cultural needs of different populations [30]. This is particularly relevant to First Nations people who face barriers to self-management which are distinct from the non-First Nations population. Programs targeted at addressing First Nations-specific barriers may improve aspects of self-management in this population [8]. Models of care must involve an awareness of the social, emotional and economic context of First Nations groups with sensitivity to the consequences of the grief and loss associated with colonisation [31]. The WHO urges action to empower people with noncommunicable diseases to manage their own condition better, through provision of tools for self-management and strengthened capacity for evaluation [2]. Implementation and evaluation of such approaches and strategies is pivotal for progress in the field, particularly for First Nations people.

There is increasing evidence for improved outcomes across physical health conditions and depressive illness in response to self-management strategies [25, 32]. However, challenges to implementation of self-management have been reported [33, 34]. These include wide variation in the nature of strategies [35], and uncertainty around preferred outcome measurement [23]. Boger and Ellis et al. reported that preferred outcomes ranged across stakeholders from knowledge, skills, and bio-psychosocial markers of health, through to ‘being me’ and having positive social networks [22]. Further challenges are posed by attempts to implement collaborative goal setting—a key factor in fostering self-management. For example, service providers’ need to shape participants' goals into pre-determined health behaviour change often takes priority over the empowering and motivating process of collaboration with service users [36]. While a wide range of self-management interventions were recently reviewed by Donald et al. (2017), patient engagement in the design of the interventions was notably lacking [7] and evidence of acceptability and feasibility of self-management approaches within First Nations communities remains scarce.

As a member state of the WHO, Australia has an international commitment to address noncommunicable diseases in line with the Global Action Plan. The Australian National Strategic Framework for Chronic Conditions recommends targeted action to facilitate individual, community and population empowerment, defining empowerment as the process by which people gain control over the factors and decisions that shape their lives through consultation and communication [37]. In Northern Australia, there is a pressing need for consultation, communication and implementation of interventions that are culturally responsive to the priorities and values of First Nations people [38]. While most First Nations people speak their own language at home, in contrast, the majority of health professionals they will meet on their health journey are non-First Nations and communicate in English [39, 40]. Requirements for holistic, culturally responsive care in Chronic Kidney Disease (CKD) have been understood and expressed through services such as ‘Purple House’ in Alice Springs (the Western Desert Nganampa Walytja Palyantjaku Tjutaku First Nations Corporation), which developed a model of care based around ‘family, country and compassion’. Along with offering dialysis, aged care and disability support services, Purple House offers support for accommodation and financial relief, transport to attend appointments, return to country visits, and social activities such as bush picnics and visits [41]. Nevertheless, gaps persist in chronic conditions care in the NT with recommendations for change including: more patient health education, broadening of health care provider perspectives (e.g. to a holistic approach), strengthening links with the community (e.g. supporting return for cultural ceremony), and adapting the service environment (e.g. to be more family friendly) [42]. What is not known is whether individualised, culturally responsive self-management interventions can assist in filling such gaps. This collaborative study between mental health and kidney disease researchers sought to answer this question.

The Aboriginal and Islander Mental Health Initiative (AIMhi) in the Northern Territory has conducted foundational work over two decades developing culturally responsive wellbeing tools through grass roots involvement with First Nations people and guidance by First Nations Expert Reference Groups [43–45] These tools take into account the ‘whole of life’ view of First Nations people where cultural, spiritual and social wellbeing are integral to health, and are informed by study findings that First Nations people in the NT prefer holistic messages using storytelling, language, art work and key cultural informants to convey health information. The AIMhi goal to promote health literacy and accessible mental health treatment led to development and testing of a new brief therapy ‘Motivational Care Planning’ [46]. Designed to address mental illness and related comorbidity through a holistic and empowering approach, the ‘Stay Strong Plan’ immediately attracted interest from colleagues in substance misuse, chronic conditions and palliative care [44, 47, 48]. The AIMhi Stay Strong care plan addresses psychosocial as well as physical needs consistent with a model of person-centred rather than patient centred care. Family and community are prioritised and the prioritising of support, strengths, worries and preferred lifestyle changes is led by the individual rather than the clinician or support worker. The AIMhi Stay Strong care plan was co- designed with First Nations People to overcome cultural and language differences using limited text in plain English, pictorial elements and relevant metaphors. In accord with the principles of trauma-informed care [49], it incorporates an empowering, strengths-based approach to collaborative goal setting [46]. Stay Strong care planning has undergone consultation, codesign and implementation cycles over a decade, including transition to a digital format in 2013, with gathering evidence of acceptability, feasibility and efficacy [44, 47, 48, 50, 51]. The transition to digital format resulted from stakeholder consultation and feedback. Further consultation and implementation highlighted the acceptability of the new interactive and visual format [52, 53]. It also aligned with the evolution of digital health care and electronic health records.

The tool was further adapted for use with people with chronic kidney disease in 2015 in the lead up to testing its effectiveness through the ‘Wellbeing Intervention for Chronic Kidney Disease (WICKD)’ clinical trial [54]. The trial aimed to determine whether the AIMhi Stay Strong app improved mental health and wellbeing for First Nations people receiving haemodialysis. Published results show significant improvement in wellbeing for participants with symptoms of depression and emotional distress at baseline [55].

This paper recognises that the effects of complex interventions are difficult to explore using quantitative methods alone [56] and examines qualitative data collected in the Stay Strong app through the course of the WICKD trial. This data includes self-identified strengths, worries, and lifestyle goals for change. While the trial also showed that the contact control app (a brief, culturally responsive health education session about Hepatitis B) improved distress and depression symptoms, that app is not included in this analysis as it focuses on sharing information, does not collect qualitative data, and does not support participants to set collaborative goals.

This study had two objectives:

1. To explore the suitability of the Stay Strong app as a self-management tool in End Stage Kidney Disease as shown by the readiness of participants to engage in goal setting.
2. To give voice to participants priorities and values as revealed through their self-management goals.

## Methods

### Participants and setting

This study examined qualitative data collected within the Stay Strong app through the WICKD clinical trial (ACTRN12617000249358). The trial methods have been detailed elsewhere and are summarised here for context [57]. Prior to commencement of the trial the Stay Strong app was adapted for use with First Nations people receiving haemodialysis through collaboration between the research team, an expert panel including those with lived experience, and the Australian Interpreter Service. The 8-member research team comprised five non-First Nations members with expertise in mental health and kidney health research in First Nations settings, a Torres Strait Islander renal physician and research fellow, and two First Nations and Torres Strait Islander research officers, one of whom spoke five Central Australian First Nations languages. A 9-member expert panel was established, consisting of two renal physicians, a renal dietician, four renal health nurses, one of whom is also Chief Executive Officer of Purple House (a First Nations-owned and operated dialysis service based in Alice Springs mentioned earlier), a cultural consultant and First Nations Elder from Central Australia, and a kidney transplant recipient. The expert panel assisted the research team in adaptation of the Stay Strong App for kidney patients. The research team worked in collaboration with the NT Government Aboriginal Interpreter Service (AIS), which has offices in Darwin and Alice Springs and employs approximately 30 interpreters. The service provides interpreting and translation for the major languages of the Northern Territory and employs a further 400 casual interpreters covering nearly 100 languages and dialects. The chosen intervention, processes and outcome measures (Kessler 10, Patient Health Questionnaire 9, and EuroQoL) were examined and adapted through 3 stages:

1. Pilot testing of feasibility and acceptability in a purposive sample of five people receiving haemodialysis treatment and carers;
2. Translation of outcome measures through collaboration between the Aboriginal Interpreter Service, First Nations research officers and the research team into 11 First Nations languages (Warlpiri, Arrernte, Luritja, Pitjantjatjara, Alayawara, Tiwi, Kriol, Yolngu Matha, Ngangikurranggurr, Murrinh Patha, Anindiliyakwa),
3. Conversion of paper-based outcome measures to electronic format

Modelling the complex intervention prior to full-scale testing provided important information about the design of both the outcome measures and the intervention [54]. These changes were designed to support success in conduct of the clinical trial and future implementation of the intervention in clinical settings. The above processes confirmed that the study addressed needs articulated by people and communities, allowed active engagement of community members in negotiations about the research topic and the methods of research and ensured fully informed consent in accord with NHMRC guidelines [58].

Following the modelling phase and completion of all adaptions to the tools and processes, the clinical trial commenced. This three-arm, waitlist, single-blind randomised controlled trial tested the efficacy of the Stay Strong App intervention in improving wellbeing among First Nations people undergoing haemodialysis for kidney failure in Alice Springs and Darwin. Data collection occurred between February 2017 and March 2019. Consent, interventions, and outcomes measures were completed by First Nations research officers accompanied by non-First Nations researchers. Pictorial information sheets and flipcharts in plain English and 11 NT First Nations languages assisted understanding. Demographic information and outcome measures were collected using a tablet device that includes pictorial prompts and First Nations language recordings for each item (choice of 11 NT languages). Interpreters were utilised where necessary. Assessment and treatment sessions occurred at a place identified by the participant as most comfortable for them: outdoors, at the health clinic, while receiving haemodialysis, or at their accommodation.

Participants were randomised to three treatment conditions. One condition (‘immediate treatment’) received the AIMhi Stay Strong app at baseline and at three months, and the other two conditions (‘contact control/delayed treatment’ and ‘usual care/delayed treatment’) received the app at 3 months. Outcome measures were completed at baseline, 3 months and 6 months [55]. Participants were reimbursed for their time with a supermarket voucher at each follow up point. In addition to their allocated treatment, all participants received usual care from their kidney service.

Participants were First Nations Australians aged ≥ 18 years, receiving maintenance haemodialysis in Alice Springs or Darwin for more than 6 months. Exclusion criteria were an age < 18 years, having visual impairment, a current guardianship order, or being otherwise unable to provide informed consent. Participants were recruited into the study following face to face invitation whilst attending their usual haemodialysis care services. The appropriate time point for recruitment post commencement of haemodialysis was given detailed consideration within the research team. The decision to exclude those receiving haemodialysis treatment for less than six months took into account expert opinion that the first six months may represent a time of particular distress, and that the initial negative impact of this life change may then settle to some extent. There was nevertheless recognition that distress will change further over time. The study sought to test effectiveness of a wellbeing intervention and aimed to minimise such confounding effects where possible. That there is such a change following commencement of haemodialysis is supported by reports from First Nations people in rural Australia of a journey from ‘shock’ to ‘acceptance’ and a ‘more positive attitude’ following commencement of haemodialysis [59]. This time line also accords with findings by Moore et al. (2020) of initial worsening followed by improvement in Quality of Life (QoL)12 weeks after starting dialysis [60], while others have reported that QoL deteriorates as duration of dialysis increases [59, 61]. Access to haemodialysis at home was another important demographic factor discussed within the research team. It was deemed likely to influence wellbeing positively and hence a carefully worded question allowed participant perspectives of ‘access’ and ‘home’ to be recorded, recognising that some people identified home as their remote community whilst others may identify it as the urban setting in which they were interviewed. This research team decision was similarly supported by reported First Nations people’s experience [59].

As expected, baseline symptoms of depression and distress were common, with 45% (70/156) of participants scoring in the moderate/severe range on the chosen depression screening tool, the Patient Health Questionnaire-9 (i.e. >  = 10) and 39% (61/156) scoring in the moderate/severe range on the chosen emotional distress scale, the Kessler 10 (i.e. >  = 25) [55]. Consistent with the high mortality and morbidity experienced amongst the dialysis population, nine participants died during the study for reasons unrelated to the trial, and two were withdrawn due to being too ill to participate.

### Consent, ethics and funding

Approvals were granted by the Central Australian Human Research Ethics Committee (CAHREC No: HREC-16–406) and the Human Research Ethics Committee (HREC) for the NT Department of Health and Menzies School of Health Research (HREC-16–2599), which includes a First Nations subcommittee. Fully informed oral consent was obtained from all participants using pictorial information sheets and flipcharts in plain English with First Nations language versions available. Demographic information and outcome measures were collected using a tablet device including pictorial prompts and First Nations language recordings (choice of 11 NT languages). Interpreters were used where necessary. This study was supported by the National Health and Medical Research Council (NHMRC) project grant (GNT# 1,098,311).

### Data collection

#### The intervention

The Stay Strong intervention was delivered by a total of 15 researchers based in Alice Springs and Darwin (13 female, 2 male) one of whom is an author of the paper (MS). Eight (53%) had clinical qualifications (occupational therapy, nursing, Aboriginal Health Worker, psychology and naturopathy), and eight (53%) were First Nations people of whom all had experience of family members living with chronic conditions. Effort was taken to ensure continuity throughout the intervention to maximise engagement and trust, whilst ensuring follow up measures were conducted by alternative researchers blinded to treatment condition. None of the researchers were involved in delivery of care to participants although some (approximately 4) researchers delivering the intervention were known to participants prior to commencement through kinship or social connection. Interaction with participants outside of the intervention was rare; but occasionally First Nations researchers interacted with participants external to the research setting (linked with social connections in the relatively small communities of Darwin and Alice Springs). Following Stay Strong training by experienced trainers within the research team (TN, MS), the intervention was delivered in teams of two (one non-First Nations and one First Nations researcher), consistent with the AIMhi Stay Strong Planning Brief Treatment Manual [21]. Reviews of app data and ongoing booster training sessions provided feedback to these researchers, allowing adjustment to their mode of delivery as needed [16]. Participants were purposively recruited following referral from the treating team to the clinical trial and were initially approached face to face in haemodialysis settings. The interviews involved participant and two researchers only and were usually held in haemodialysis waiting areas or homes or hostels, while for a few, the intervention was delivered during haemodialysis.

The Stay Strong app brief intervention, designed to be delivered as a 20-min session, embodies key elements of a face-to-face semi-structured interview. It incorporates a series of prompt points: family (who supports you?), strengths (what keeps you well?), worries (what takes your strength away?), and strategies for change goals/needs (what goal for change would you like to make and why would that be a good change?).

Each of the prompt points is supported by colourful representative images with twelve choices each for strengths and worries organised under four categories, with the option to add others as desired (Table 1). At each step of the session, responses were entered concurrently, allowing the participant to guide the input of their data during the session. Motivation was enhanced through direct comparison between strengths and worries. This promoted discrepancy, a key element of motivational interviewing [62].

**Table 1:** Stay Strong app prompts prior to goal setting

**Things that keep me strong**			
Spiritual and cultural	Physical	Family, social, work	Mental and emotional
Cultural Identity	Health centre	Work or jobs	Understanding health
Connection to culture and country	Healthy food	Teach kids	Music and dance
Obligation	Exercise	Family and friends	Think strong way
Other	Other	Other	Other
**My worries**			
Spiritual and cultural	Physical	Family, social, work	Mental and emotional
Cultural identity	Being sick	Family worry	Too worried or sad
Missing cultural and country	Unhealthy lifestyle	Gambling	Mixed up thoughts
Obligation	Gunja, grog, smokes	Anger or violence	Hearing voices
Other	Other	Other	Suicide and self-harm

The interview was designed to assist in establishment of rapport through sitting side-by-side to view the shared screen, thus avoiding direct eye contact if preferred. A further design element to strengthen therapeutic alliance was the discussion of relationship and strengths prior to exploration of concerns. In addition, the training emphasised that direct questions can be experienced as challenging. Instead, practitioners were encouraged to show the relevant images and simply ask, for example ‘Are any of these worries for you?’.

The training also taught the importance of setting goals which were specific, measurable, accessible, relevant and timely (SMART), in line with recommendations in the literature [63, 64]. It highlighted that to maximise motivation the participant was to choose their own goals, while the role of the researcher/therapist was to facilitate their decision making rather than to mould it. The app supported successful goal setting through exploring steps to the goal, covering what would be done, when it would be done and who might support that step. A maximum of two goals was encouraged during each session. At goals review, feedback about progress was given and previous goals might be kept, revised, or replaced. The session concluded with development of a pictorial summary which was reviewed prior to completion. The summary was then printed and delivered to the participant. The review of the plan and delivery of the personal summary was designed to enhance motivation, whilst also allowing participants to review their inputted information.

The interview took approximately 20 min using the AIMhi Stay Strong app, with a second session of the same length delivered within 2–4 weeks that was also guided by the app. Session 1 explored family, strengths, worries and goal setting. Session 2 reviewed information entered previously, refined the goals and addressed any barriers to goal attainment, setting new goals as appropriate. Participants received a text message or phone call one week following the initial treatment reminding them of their goals and steps for making changes.

### Data analysis

Qualitative data from app sessions were analysed using a combined quantitative and qualitative content analysis approach, guided by Graneheim and Ludman [65]. Content analysis had its beginnings in the seventeenth century as a process of systematic analysis of text and by the early twentieth century was formally developed as a method in the social sciences. It transformed over time from simple analyses of the frequency of certain key words or themes in forms of communication such as hymns or newspapers, to a research technique for the objective, systematic, and quantitative description of the manifest content of communication. Further development through its use in many diverse disciplines led to the distinction between quantitative and qualitative content analysis. The focus of qualitative content analysis is on latent meaning, meaning that is not immediately obvious, whereas quantitative content analysis focuses on manifest, literal meaning. There is, however, no sharp line dividing quantitative and qualitative content analysis as is demonstrated in the analysis undertaken in this paper.

**Fig. 1:**
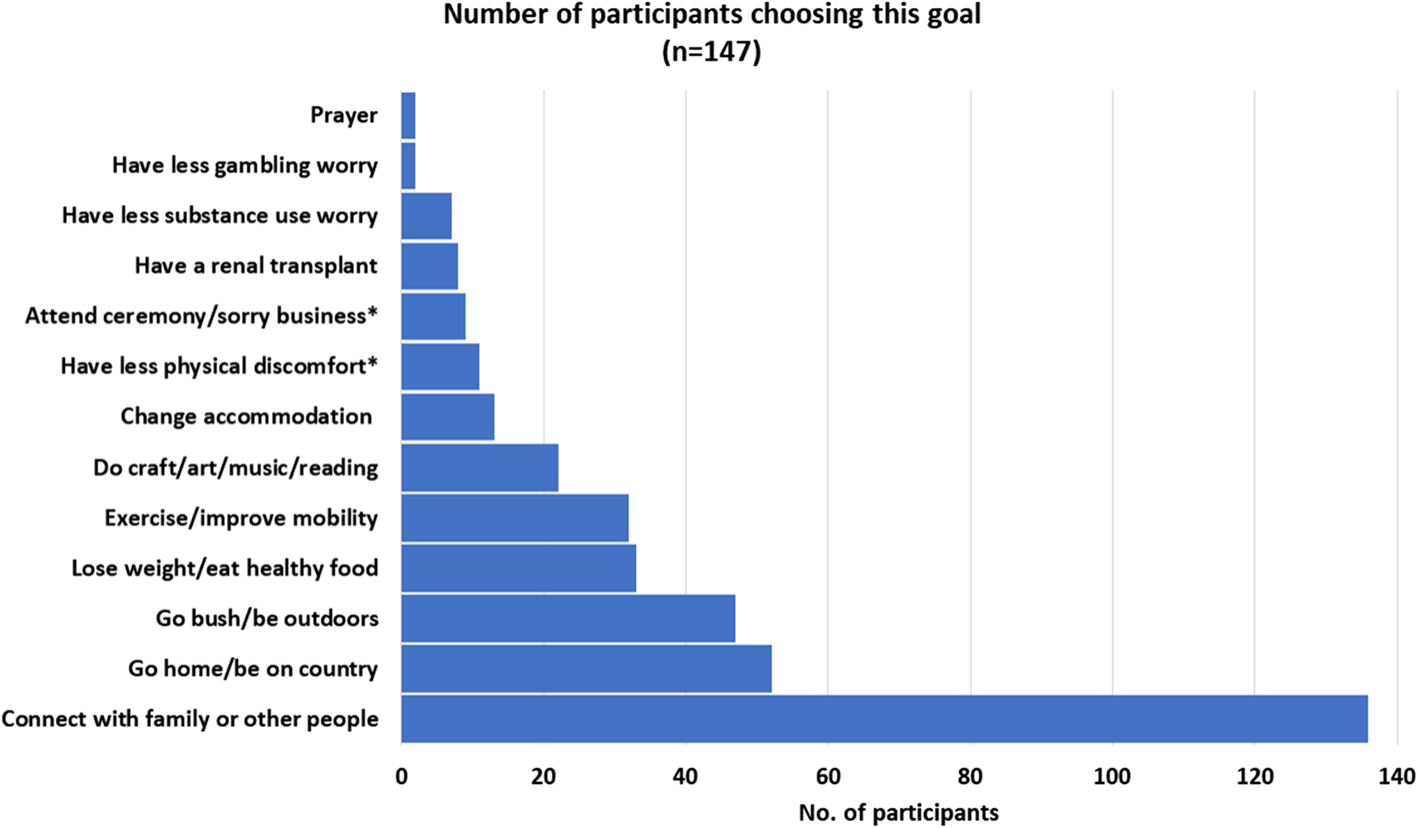
Participant Goal Categories. * Types of physical discomfort included worry about eyes, sleep, teeth, pain, and dressings. * Ceremony refers to gatherings that reflect the diversity of cultural and spiritual practices within communities and that help pass down this rich cultural knowledge.* Sorry business refers to cultural practices and protocols associated with death

We used content analysis as a descriptive research method through development of a coding frame and qualitative coding of data. The coding frame was both concept driven (defined in advance through the structure of the Stay Strong app) and data driven (derived from data during coding). The initial identified unit of analysis was each individual response to the goal setting section of the interview presented in the words of the participant. This section explored goals for change and reasons for making that change. These responses were recorded verbatim in the app as participants responded and later uploaded to the study data base. Prior to analysis the data set was reviewed, and goals that were repeated in later sessions were removed so that each goal was only included once. Using a deductive approach to manifest content, manual open coding of the data led to identification of 13 categories following which their frequency was counted (Fig. 1). In a subsequent step, an inductive approach (with greater focus on latent meaning) allowed identification of broader themes. Codes, categories and themes were first discussed between the male Senior First Nations Cultural Consultant to the study (PJM) and TN (female, psychiatrist and senior principal research fellow), and were then checked with female research colleagues (MS and KD) until consensus was reached. Opportunity for participant (member) checking of interview responses occurred during the original session as participants watched or assisted in initial data input, and again at the stage of generation of the app ‘summary’ which occurs at the end of the session and summarises the inputted information, and once more at the time of sharing the printed summary from the session (usually immediately following the session). Elements supporting trustworthiness of the analysis thus include the accuracy of data collection via electronic input into the app intervention through collaboration between researchers and participants, the member checking steps integral to the intervention, description of coding steps, and peer checking. Data saturation appeared to be reached as comments were noted to be similar and repeated with little new information apparent toward completion of the analysis. Our own role as researchers in collection of such data requires acknowledgement. Although viewed in this paper as a ‘semi structured interview’, the intervention was a component of a clinical trial. As such it was manualised, intervention training was delivered to all researchers, and fidelity of intervention delivery was regularly reviewed. Field notes were not used to separately describe and record the intervention/interview process. The Stay Strong approach emphasises the importance of allowing participants to lead responses. Participants are seen as the experts in their lives and researchers are encouraged not to offer suggestions but to facilitate participant thinking about each step of the intervention. Although invariably the researchers own biases will have influenced responses through data collection, the complex design of the study requiring 15 different researchers may have minimised a persistent pattern of bias. At the time of analysis, the data were already collected in a data pool, however theoretical assumptions during interpretation will also have influenced the thematic analysis. The female non-First Nations first author and First Nations consultant have worked together in the AIMhi program for twenty years. Interpretations are likely to have aligned with our life and research experience, for example our previous research findings and related beliefs that preference empowerment, holistic views of wellbeing and the importance of cultural values.

## Results

### Demographics

The total number of participants entered into the trial were 156. Sixty-two participants were allocated to the immediate treatment condition, 61 to the contact control/delayed treatment condition, and 33 to the treatment as usual/delayed treatment condition. Thus, all participants had an opportunity to receive the Stay Strong intervention at some stage. Data collection occurred between February 2017 and March 2019 in Darwin or Alice Springs. Most participants were female with a mean age of 55 years, had been receiving haemodialysis for more than three years, and did not speak English as their first language (Table 2). Of those meeting inclusion criteria (181), 25 declined to participate.

**Table 2:** Participant demographics

Gender	Male	N (%)	44 (28%)
	Female	N (%)	112 (72%)
Age (in years) at randomisation		Mean (SD)	55 (9.4)
Years since initiation of haemodialysis		Median [IQR]	3.1 [2.0–5.7]
English as first language		N (%)	28 (18%)
Access to dialysis at home community		N (%)	97 (62%)
Top End		N (%)	78 (50%)
Central Australia		N (%)	78 (50%)

### Interventions received

Of the 156 participants recruited to the study, 147 (94%) participated in Stay Strong sessions. Those who did not receive a session were too tired, too unwell, had moved away or had passed away. Of the 147 participants who received a Stay Strong session, 12 (8%) received only one session, 80 (54%) received two sessions, 14 (10%) received three and 41 (28%) received 4 sessions. Most sessions were delivered as planned at each time point, and the average length of a first session ranged from 22–24 min and average length of a second session ranged from 14 to 19 min [55].

### Participant goals

In total, 262 separate goals, together with reasons for choosing that goal, were entered into the app. Most participants (83/147, 56%) set more than 1 goal: 56 (38%) chose two goals, 24 (16%) set three and 3 (2%) set more than three goals (M = 1.8). Many separate goals incorporated more than one type or category of goal, for example a goal to ‘connect with people’ often also included a goal to ‘go home’ or ‘to country’, and a goal to ‘exercise’ often included plans to ‘go hunting’. Exploration of goal data through content analysis resulted in 373 individual goals and 13 distinct but interlinked categories. The most frequent of these was to ‘connect with family or other people’ (Fig. 1). The incorporation of more than one category into a goal is illustrated in the sample responses in Table 3.

**Table 3:** Sample responses from each participant goal category

Connect with family or other people
• See more family from out of town to feel more connected and less disconnected from country • Keep credit on my phone so I can call my family and friends • Arrange women’s meeting regularly when not on dialysis days [so I can] have a yarn with other ladies, make craft, feel productive like in Broome WA (Western Australia) • Get my Wi-Fi back on [so I can] talk to my family via Facebook, play games again
Go home be on country
• Go back and see country [so I can] do fishing, camping, watching sea gulls, drinking tea, going hunting • Go back to community and eat good bush tucker* [so I can] feel good and happy to be on country and see family
Go bush/be outdoors
• Go hunting, get some bush tucker* [so I can] sit down and listen to birds, get bush tucker, get out and cook bush potatoes • Go turtle hunting with family [to be] feeling good, happy when go with family
Lose weight/eat healthy food
• Come to exercise group at dialysis or Danila Dilba* [because its] good for body, losing weight, feel healthy, get out of house • Go dig for yams [because] bush tucker* is good for body
Exercise/improve mobility
• Be able to walk again with prosthetic leg to work my body again, to get healthy • Get more exercise [because it will] make me strong and healthy so can feel good about myself
Do craft /art/music/reading
• Do some weaving, make mats, dilly bags* [because its] connection with culture • Do shell painting with Tiwi Children [so I can] teach young one’s culture, feel good
Change accommodation
• Find somewhere to live away from hospital, get outside [so I can] be happy, see more family • Move out of hostel [because] I’d feel safer and will be with family)
Have less physical discomfort
• Get bush medicine* [so I can] stop itching and feel more relaxed • Get eyes fixed-so I can see better, feel more independent
Attend ceremony/sorry business
• Go home for funeral [so I can] pay respect, support family • Go to APY homelands* for funeral [so I can] see family, pay respect, say goodbyes
Have a kidney transplant
• Stay healthy for transplant [so I can] go back home • Get a transplant to live a normal life and travel
Have less substance use worry
• Think about quitting smoking for my kidneys and health • Stop drinking grog [to have] no more headache or stomach pain, better sleep, better walking
Have less gambling worry
• Not use all my money in gambling so I can have some for shopping

^*^Bracketed additions represent prompts within the app

^*^Bush food or bush tucker is any food native to Australia taken from land and sea and used as sustenance by First Nations Australians

^*^Danila Dilba is an Aboriginal community-controlled organisation providing comprehensive primary health care and community services to greater Darwin region of the NT

^*^The dilly bag is a traditional string bag, made by First Nations people from twisted bark fibres used for carrying personal and medicinal items and sacred artefacts.*Ancient and traditional bush medicine is typically prepared from Indigenous flora and fauna and earth (salt, clay, minerals), for spiritual and physical healing

^*^Aṉangu Pitjantjatjara Yankunytjatjara (APY) Lands, is a large, sparsely-populated area for First Nations people, located in the remote north west of South Australia

All participants who set goals then completed the step setting component of the app, with the vast majority setting more than one step toward at least one goal (133/147, 90%). Goals were linked with steps which were generally specific, measurable, accessible, relevant and timely, as prompted within the app and as demonstrated in Table 4 below.

**Table 4:** Examples of goals with addition of the steps chosen*

[I want to] lose some weight for transplant [because it] helps for dialysis [so the first thing I will do is] buy boots on Wednesday [with help from] (person’s name) [and the second thing I will do is] start walking from home to gorge—4 km [and another thing I will do is] talk to nurses about dietician
[I want to] do painting or make dilly bag [so I can be] less bored [so the first thing I will do is] get canvas and paint next week [and the second thing I will do is] ask mother in law to bring in pandanas* [with help from] my husband this week
[I want to] help young people so young ones can care for old ones [so the first thing I will do is] bring them out bush regularly during school break [with help from] other women in community [and the second thing I will do is] teach them how to care for themselves [and another thing I will do is] teach them songs and drumming

^*^Bracketed additions represent prompts within the app

^*^Pandanus leaves are traditionally used by First Nations peoples for weaving baskets

**Fig. 2:**
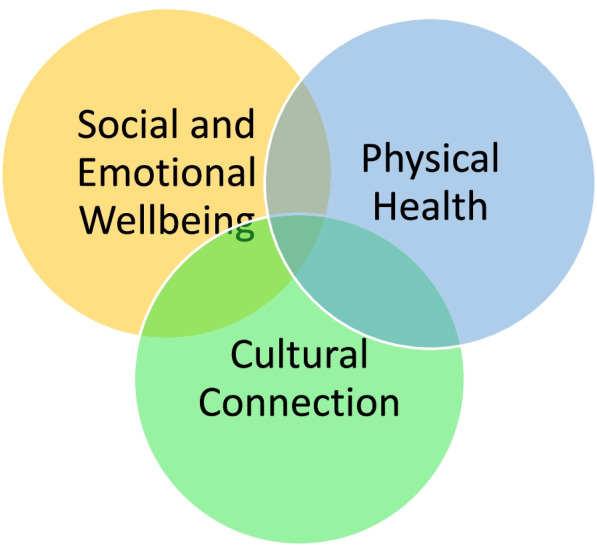
Three broad themes within participant goal setting

Further examination of latent content within the qualitative data identified three broad themes: ‘physical health’, ‘cultural connection’ and ‘social and emotional wellbeing’ (Fig. 2). Examples of goals which demonstrate these themes are given in Table 5. The overlap of these three themes was visible throughout the vast majority of the responses. The examples shown in Table 3. provide further demonstration of these themes with frequent references to feeling ‘good’, feeling ‘happy’ or feeling ‘relaxed’ linked with actions addressing physical health or cultural connection.

**Table 5:** Examples: physical health, cultural connection, social and emotional wellbeing themes

Physical health linked with wellbeing
• Want to do renal training to do selfcare [so I can] get back home and family happy for me • Attend last dentist appointments [so I can] finally be on transplant list
Social and emotional wellbeing linked with family / cultural connection
• Talk with friends share stories and food [because it] makes me happy • Find somewhere to live away from hospital, get outside [so I can] be happy, see more family
Cultural connection linked with physical health
• Talk to family about country not drinking grog [because it’s] good to talk about country and tell stories and sharing of dreamtime with kids. My grandfather did this in Nyrripi [NT community in Central Desert Region] • Go to APY homelands for funeral [so I can] see family, pay respect, say goodbyes

## Discussion

The health disparities existing between First Nations peoples and non-First Nations populations require urgent attention. Health systems and models of care need to be contextualised for First Nations communities [3]. Supporting people to self-manage their own condition is key, along with promotion of cultural heritage and traditional knowledge and empowerment of First Nations communities and leadership. There is need to transform medical models of care and transition to person-centred care paradigms that preference social context and empower individuals with knowledge and strategies and autonomy of choice. These must also adapt to many varied contexts and be evaluated in terms of implementation and effectiveness. This study demonstrates the potential for a culturally responsive tool, already found to have high acceptability and feasibility among First Nations people, to be used in provision of a person-centred approach to self-management. The tool has developed iteratively through systematic collaborative research over two decades, and recent adaption and pilot testing to the chronic conditions setting [54].

Despite physical illness, low mood, and high distress, dialysis participants engaged actively in this self-management intervention. The vast majority chose to attend follow up sessions, most chose more than one goal, and most chose more than one step to that goal. The goals chosen aligned with the holistic view of health of First Nations peoples documented elsewhere, with connection to culture, land and family interwoven throughout [46, 66]. The top three categories were: ‘connection with friends or family’, ‘going home/being on country’ and ‘going bush/being outdoors’. Social connection was chosen more than twice as often as other goals. This need for social contact and family connection, and preference for treatment at home or visits home (often to their remote community) is well aligned with established evidence of the sense of isolation and displacement from family, country, and identity caused by relocation for dialysis [67].

The engagement and alignment of goals with recognized values suggests acceptability and suitability of the intervention in this setting. This suggestion is strengthened by the findings of the clinical trial that those with high distress or depression scores at baseline showed clinically significant improvement at 3 and 6 months follow up [55]. On the other hand, the clinical trial showed no impact on haemodialysis attendance [55]. This result accords with systematic review findings of limited and inconsistent impact of self-management on medical outcomes, while mental illness is among the conditions with best evidence of effectiveness [68]. The findings affirm the importance of the holistic models of care offered through services such as Purple House (‘family, country and compassion’), and the significance of continuing to promote mobile dialysis services and dialysis clinics on country [42, 66]. They also suggest that shaping the goal setting of clients by service providers to ensure physical health needs are addressed is unwarranted [36], since participants were clearly aware of their own physical health needs. The need for a holistic approach to their needs was exemplified by their choice of self-management goals that intertwined physical health needs with social and cultural priorities. This finding is not surprising given that First Nations peoples define wellbeing far more broadly than merely physical health, and that connections with land, language, family and identity are essential components of wellbeing [69]. In addition, the findings highlight a range of simple interventions that CKD outpatient clinics and services working with similar client groups might prioritise, such as access to: phones, WIFI, radio, music, creative opportunities such as painting and weaving, fitness and mobility training, assistance with health care appointments and diarising, along with family-friendly welcoming environments as recommended in best practice guidelines [70].

The emphasis on sociocultural priorities in goal setting contrasted starkly with the relatively few goals chosen to directly address physical complaints (sleep, pain), substance use (alcohol smoking, and cannabis) or specific mental health issues, for example through counselling. On the other hand, the interdependent nature of all the categories is evident, with mobility, for example, strongly linked with outdoor activity and going to country, as well as to physical health and social and emotional wellbeing. Although not directly addressed, it is therefore likely that these specific health issues were indirectly addressed through other goals. The relatively few participants who chose a goal specific to mental health or counselling might be interpreted in different ways. In terms of the interdependence of the categories it is likely that social and emotional wellbeing needs are perceived to be met by activities such as social and cultural connection. On the other hand, it might be explained by concerns about cultural safety [71], or by low accessibility of culturally appropriate mental health services [72]. Whichever of these possible reasons is paramount, the need for SEWB intervention is clear given the high comorbidity of CKD with distress and depression [73].

Integrating SEWB and chronic disease treatment is an important strategy in this cohort where physical illness and disability combine with limited access to transportation [37]. The findings of this study support suitability of this tool as a combined chronic condition self-management and social and emotional wellbeing intervention. Additionally, it gives voice to the individual needs of consumers, allowing services to review and adapt their processes in the light of identified consumer priorities.

### Limitations

One limitation of this study is that the data included multiple sessions for some individuals and single sessions for others. Thus, some individuals are overrepresented compared with others. While repeated goals were not included multiple times, multiple sessions per participant were included to inform the range of goals that participants set. Another potential limitation is that researchers may have coached participants in their goal setting despite the protocols and training in place to seek to avoid this approach. The findings, however, show goals that align well with findings from similar work in the field, suggesting that any such bias was not systematic [42, 46]. Another limitation to consider is that the description of the goals is relatively ‘thin’, with little detail providing context of the full interview. However, each of the goals were determined following therapeutic engagement in a four-step process exploring important family and friends, strengths and worries, prior to goal setting. Further, while brief in words, many of the goals evoke visual pictures bringing relationships, places and interactions to life in accord with definitions of thick description [73]. The original development of the tool in the Northern Territory, and its recent adaption to the local kidney disease setting, may limit its transferability to other regions of Australia, or to use with First Nations people in other countries. The principles of respectful collaborative research, promotion of cultural heritage and traditional knowledge, empowerment through person centred care, and robust evaluation, however, are directly transferable.

## Conclusions

In conclusion, this study demonstrates that the Stay Strong app can be considered for use as a chronic condition self-management tool for First Nations people. This result is important as there are few, if any, culturally responsive self-management support tools currently available. The findings confirm that while biomedical models must focus on the mechanics of illness, First Nations people also prioritise social connection, family, country, and cultural identity. This study is a component of a robust evaluation of effectiveness of the impact of the Stay Strong app on depression and wellbeing of First Nations people receiving haemodialysis. Additional work is needed to evaluate individual self-management outcomes such as empowerment, quality of life and achievement of health-related goals.

Meanwhile, systematic implementation of person-centred self-management strategies into routine care remains a key challenge and further research is needed to establish drivers of success.

## Data Availability

The datasets generated and/or analysed during the current study are not publicly available due to participant confidentiality but may be available from the corresponding author on reasonable request.
